# Extremely late‐onset chronic graft‐versus‐host disease presenting with bronchiolitis obliterans and pleuroparenchymal fibroelastosis

**DOI:** 10.1002/rcr2.1201

**Published:** 2023-07-31

**Authors:** Sae Akiyama, Chisa Murayama, Saori Aizawa, Arei Mizushima, Yukiko Maeda, Natsuko Taniguchi, Katsura Nagai, Toshiyuki Harada

**Affiliations:** ^1^ Center for Respiratory Diseases Japan Community Healthcare Organization Hokkaido Hospital Sapporo Japan

**Keywords:** bronchiolitis obliterans, graft‐versus‐host disease, haematopoietic stem cell transplantation, late onset, pleuroparenchymal fibroelastosis

## Abstract

A 35‐year‐old woman experienced left back pain after a 2‐h flight. She reported coughing and left back pain 1 day later when she presented to our hospital. Chest computed tomography showed pneumothorax of the left lung, bronchiectasis, thickening of the bronchial wall, nodules, and cavity lesions in both lungs. A pulmonary function test revealed obstructive ventilation disorder with normal lung diffusing capacity. She had a history of haematopoietic stem cell transplantation (HSCT) at 2 years and 3 months of age during the second disease remission of acute myeloid leukaemia. She was diagnosed with chronic graft‐versus‐host disease (cGVHD) presenting with bronchiolitis obliterans (BO) and pleuroparenchymal fibroelastosis (PPFE). To our knowledge, this is the first reported case of BO and PPFE diagnosed more than 30 years after HSCT.

## Graphical abstract

We present the case of 35‐year‐old patient, with a history of haematopoietic stem cell transplantation (HSCT) at 2 years and 3 months of age during the second disease remission of acute myeloid leukaemia, who was diagnosed with chronic graft‐versus‐host disease (cGVHD) presenting with bronchiolitis obliterans (BO) and pleuroparenchymal fibroelastosis (PPFE). To our knowledge, this is the first reported case of BO and PPFE diagnosed more than 30 years after HSCT.

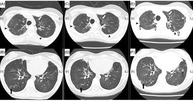

## INTRODUCTION

Haematopoietic stem cell transplantation (HSCT) is being performed more frequently to manage various hematologic malignancies. Pulmonary complications remain a major concern after HSCT and occur in 25%–50% of patients undergoing HSCT, accounting for approximately 50% of transplantation‐related deaths.^1^ Bronchiolitis obliterans (BO) and pleuroparenchymal fibroelastosis (PPFE) are known pulmonary complications caused by chronic graft‐versus‐host disease (cGVHD) after HSCT. We present a patient with extremely late‐onset cGVHD presenting with BO and PPFE.

## CASE REPORT

A 35‐year‐old woman who never smoked experienced left back pain after a 2‐h flight and reported coughing 1 day later. She had a history of acute myeloid leukaemia (French‐American‐British categorization type M1) at 1 year and 8 months of age and received an allogenic bone marrow transplant from her older sister at 2 years and 3 months of age. Methotrexate was administered to avoid GVHD. At 3 years of age, a rash consistent with stage I acute GVHD as well as dry mouth and hypolacrimation consistent with severe chronic GVHD developed.

**FIGURE 1:**
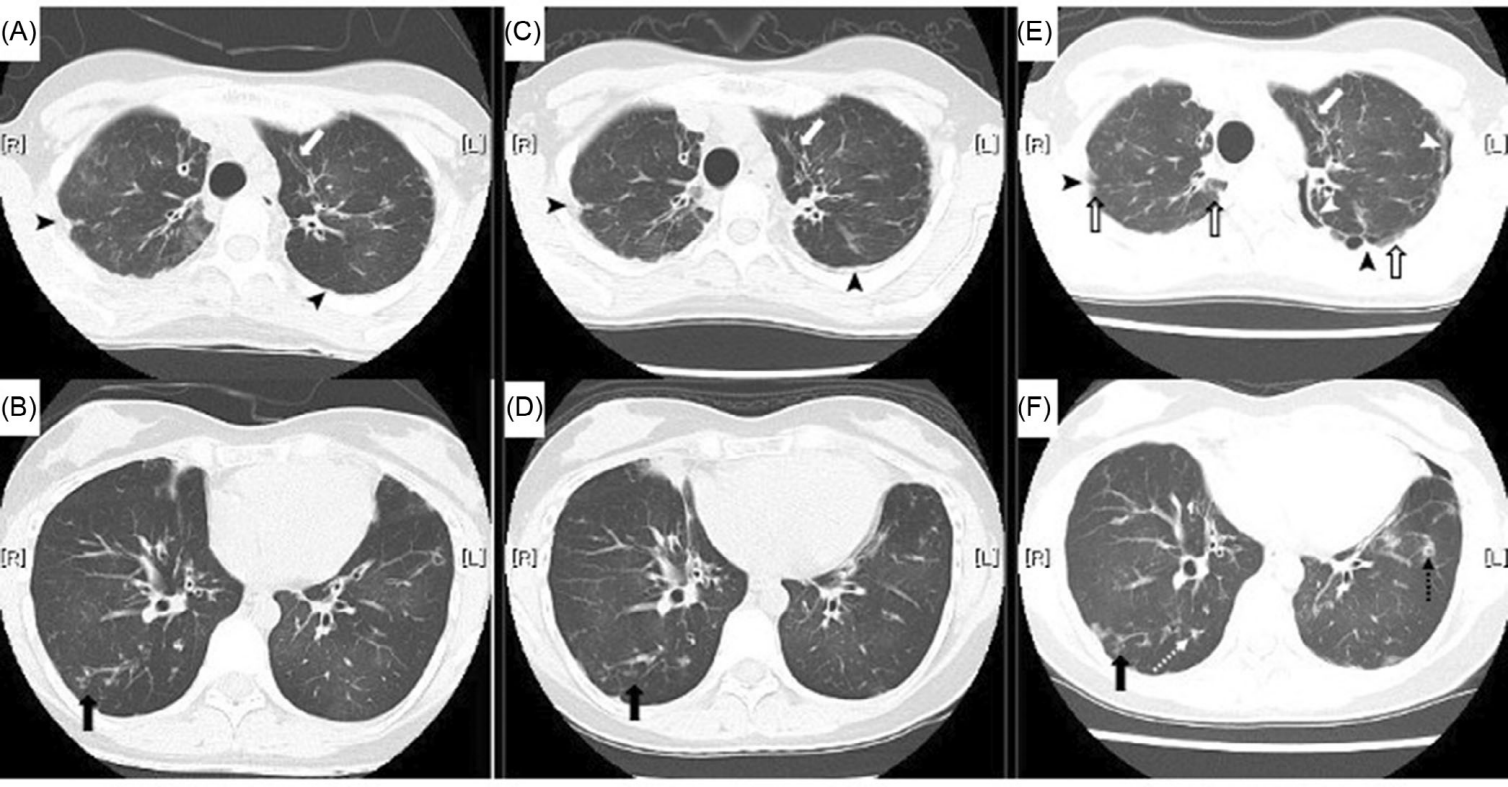
Computed tomography of the chest reveals a centrilobular granular shadow (black arrow), bronchiectasis (white arrow), and pleural thickening (black arrowhead) of the apex of both lungs at 3 years before presentation (A, B), slowly progressive bronchiectasis and pleural thickening at 2 years before presentation (C, D), and interstitial pneumonia of the upper lobe of both lungs (open arrow), pneumothorax (white arrowhead) in the left lung, bronchiectasis, bronchial wall thickening (dashed black arrow), and nodules (dashed white arrow) in both lungs (E, F).

**FIGURE 2:**
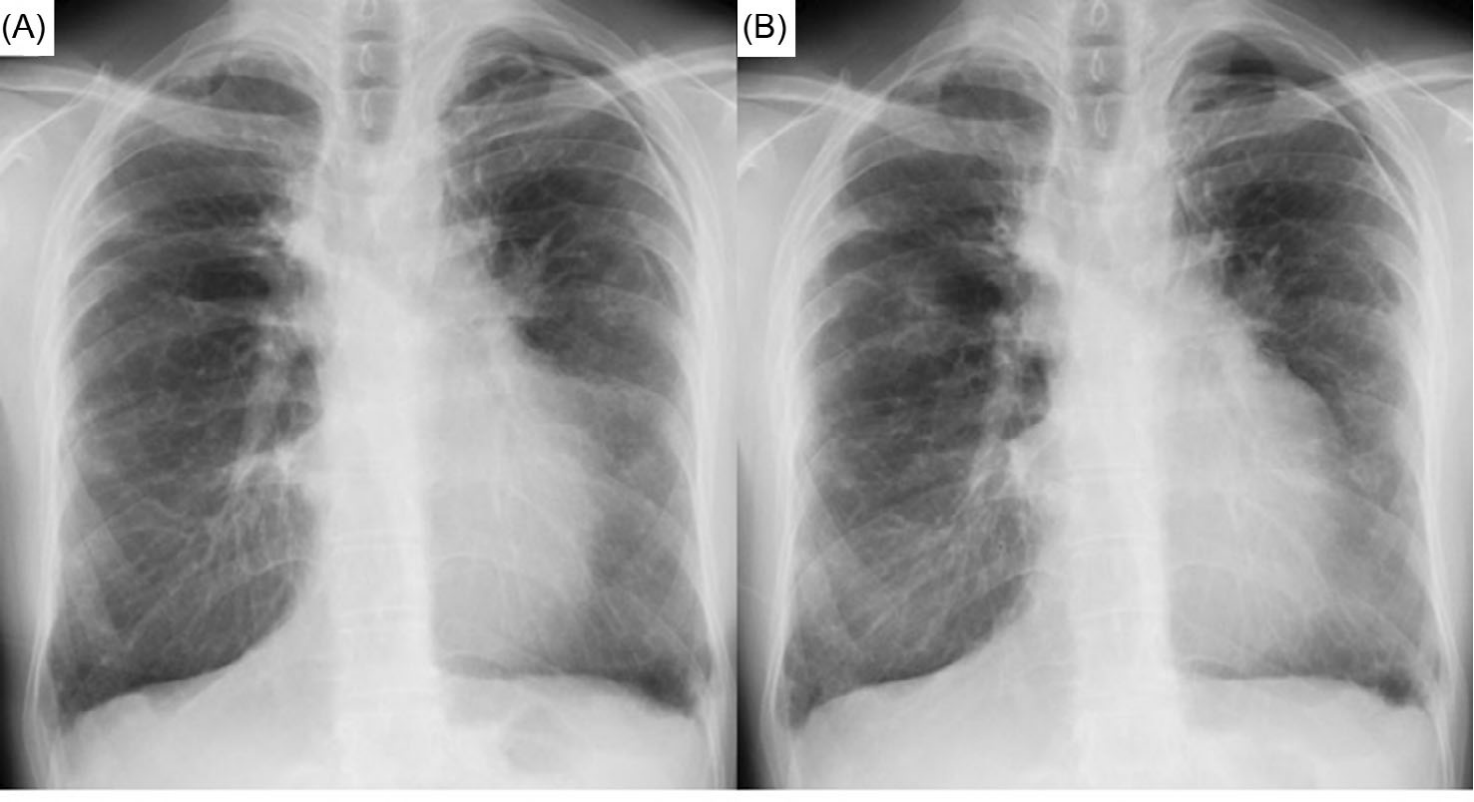
Chest radiography reveals pneumothorax of the left lung at 1 year before presentation (A) and recurrent pneumothorax in the left lung and an infiltrative shadow outside both lungs (B).

Three years before the current presentation (age, 32 years), she had pneumonia in the right lung that was successfully treated with ceftriaxone sodium hydrate; however, no causative bacteria were identified. Chest computed tomography (CT) revealed centrilobular granular shadows, bronchiectasis, and pleural thickening of the apex of both lungs (Figure 1A, B). Two years before the current presentation (age, 33 years), CT showed slow progression of bronchiectasis and pleural thickening (Figure 1C, D). Pulmonary function test results indicated the following: forced vital capacity (FVC), 1.66 L (56%); forced expiratory volume in 1 s (FEV1), 1.04 L (40%); absent bronchodilator response; and FEV1/FVC, 63%. One year before presentation (age, 34 years), she experienced pneumothorax of the left lung (Figure 2A) that improved during follow‐up without chest tube drainage. Leukaemia recurrence and intercurrent respiratory illnesses were not observed before presentation to our hospital.

At presentation, the physical examination revealed a respiratory rate of 12 breaths/min, oxygen saturation of 96% on room air, and clear lungs. Laboratory test results including interferon‐gamma release assay results were within the normal ranges. Pulmonary function test results were as follows: FVC, 1.48 L (51%); FEV1, 0.95 L (37%); absent bronchodilator response; FEV1/FVC, 61%; total lung capacity, 2.51 L (66%); residual volume, 1.05 L (96%); and diffusing capacity for lung carbon monoxide, 12.5 mL/min/mmHg (75%) after correction for haemoglobin. Chest radiography revealed pneumothorax of the left lung and an infiltrative shadow outside both lungs (Figure 2B). Chest CT showed pneumothorax of the left lung, bronchiectasis, bronchial wall thickening, nodules, and cavity lesions in both lungs (Figure 1E, F). We considered performing a surgical lung biopsy to determine the definite pathological diagnosis; however, we decided not to because of her low pulmonary function. Extremely late‐onset cGVHD presenting with bronchiolitis BO and PPFE was clinically diagnosed.

Because of her mild pneumothorax and absence of hypoxia, we planned to perform careful observation without chest tube drainage. Two weeks later, her symptoms improved and pneumothorax disappeared.

## DISCUSSION

BO is accompanied by irreversible fibrotic obstruction of the bronchioles. BO was suspected based on the pulmonary function test finding of new‐onset airflow obstruction. Pathological confirmation is rarely necessary for clinical practice. The National Institutes of Health workshop for the diagnosis of BO after HSCT criteria are as follows: FEV1/FVC <0.7 and FEV1 < 75% of predicted; evidence of air trapping, small airway thickening, or bronchiectasis on high‐resolution chest CT; residual volume >120% or pathologic confirmation of constrictive bronchiolitis; and absence of respiratory tract infection confirmed by investigations targeting clinical symptoms, such as radiologic studies or microbiological cultures.^2^

PPFE predominantly in the upper lobes is a rare form of interstitial pneumonia. Lung biopsy is not recommended for advanced cases or patients with poor ventilatory reserve, such as lung transplant recipients and HSCT patients. Thus, a definite diagnosis of PPFE could be determined based on pleuroparenchymal thickening associated with subpleural fibrosis, traction bronchiectasis, moderate reticular abnormalities, and superior involvement in the upper lobes and lesser or no involvement in the lower lobes.^3^

Because of the absence of causes of BO and PPFE other than HSCT, we diagnosed extremely late‐onset cGVHD based on the pulmonary function test and radiological results. Patients who have undergone HSCT may require routine pulmonary function tests and radiological examinations for the early diagnosis and treatment of BO and PPFE. The time from transplantation to the diagnosis of BO ranges from 2 to 16 years, and that for PPFE ranges from 3 months to 16 years.^4^ The onset of BO and PPFE as cGVHD more than 30 years after HSCT is extremely rare, thus making this a valuable case.

Systemic treatment should be considered according to the cGVHD severity classification provided by the National Institutes of Health consensus development project criteria.^5^ The standard first‐line treatment for cGVHD is corticosteroids (1 mg/kg/day). We considered this treatment option for BO and PPFE as cGVHD in this setting but did not use corticosteroids because of the following reasons: extremely late disease onset; immediate improvement after pneumothorax; mild respiratory symptoms; and the patient did not agree to this treatment because of its major side effects. If pneumothorax recurrence is not observed for a certain period, then systemic steroid treatment should be considered. We recommended a respiratory rehabilitation program to improve pulmonary function. However, her pulmonary function gradually deteriorated; therefore, she may require oxygen supplementation in the future. If her respiratory symptoms worsen, then adaption for pulmonary transplantation may be required.

## AUTHOR CONTRIBUTIONS

All authors contributed to the study conception. The literature search was performed by and the first draft of the manuscript was written by Sae Akiyama and Toshiyuki Harada. Chisa Murayama, Saori Aizawa, Arei Mizushima, Yukiko Maeda, Natsuko Taniguchi, and Katsura Nagai contributed to data curation and formal analysis.

## CONFLICT OF INTEREST STATEMENT

None declared.

## ETHICS STATEMENT

The authors declare that appropriate written informed consent was obtained for the publication of this manuscript and accompanying images.

## Data Availability

The data that support the findings of this study are available from the corresponding author upon reasonable request.
